# Structural controls on lithium mineralization in shear-zone hosted granitic pegmatites of the Zulu pegmatite field, Zimbabwe – implications for exploration

**DOI:** 10.1007/s00126-025-01371-x

**Published:** 2025-05-05

**Authors:** Lot Koopmans, Nicholas J. Gardiner, Brayden St. Pierre, Richard M. Palin, Rutendo Musinga, Laurence J. Robb

**Affiliations:** 1 Department of Earth Sciences, University of Oxford, 3 South Parks Road, Oxford, OX1 3 AN UK; 2 School of Earth & Environmental Sciences, University of St Andrews, Bute Building, Queen’s Terrace, St Andrews, KY16 9 TS UK; 3 Aure Metals Inc, 875 Exeter Street, Oshawa, ON Canada; 4 Midlands State University, P. Bag 9055, Gweru, Zimbabwe; 5 School of Geosciences, University of the Witwatersrand, Johannesburg, South Africa

## Abstract

Granitic pegmatites are a significant source of critical metals including tin, tantalum, and most notably lithium. To meet future demand, a comprehensive exploration model is required to assist in the discovery of new hard rock deposits. Whereas recent work has largely focused on understanding the source and mineralization processes of pegmatites, the structural controls on the distribution and size of individual deposits remains poorly understood and understudied. In this contribution, we present a structural study on the Zulu pegmatite field in Zimbabwe, which provides a good example of the influence of shear zones, host rock rheology, and lithological competency contrasts on the orientation, size, and distribution of pegmatite bodies within a pegmatite field. At Zulu, we observe both structural and petrographic evidence for two types of pegmatite emplacement within an active shear zone during D_2_ strike-slip dominated deformation. An early generation (Type 1) was emplaced syn-kinematic to D_2_ within dilational jogs subparallel to the shear fabric, and continued ductile shearing also drove significant recrystallization which affected the primary magmatic phases and therefore influenced the preserved mineralogy. A later generation (Type 2) was emplaced syn-to-late-kinematic to D_2_ along tension gashes and subordinate fracture sets oblique to the shear fabric, which served to truncate the cooling history and preserve a primarily magmatic mineralogy within this pegmatite group. By comparing Zulu to other large pegmatite deposits, we conclude that geologic structures are critical to source-to-sink connectivity in lithium pegmatite systems, and affect the mineralization potential of individual deposits by driving recrystallization. Assessing the structural history and relative timing of emplacement within a pegmatite field, in conjunction with detailed (micro)textural observations from within pegmatite bodies, is essential to understanding pegmatite emplacement geometries. A more systematic approach in constraining these relationships will therefore aid in generating new exploration targets in both greenfield and brownfield settings.

## Introduction

Granitic pegmatites are the primary source of several key metals critical to green technologies including tin, tantalum, and niobium, but most importantly lithium (Bowell et al. 2020; McCaffrey and Jowitt 2023). To meet projected lithium demand, pegmatite deposits are forecast to account for more than 80% of future supply (Yao 2022). Granitic pegmatites result from small-volume granitic melts through either extreme fractionation (Jahns and Burnham 1969; Černý 1991; London 2005) or low-degree partial melting of a fertile source (anatectic pegmatites) in the mid-crust (Simmons et al. 1995; Müller et al. 2017; Koopmans et al. 2024). These melts migrate upwards to be emplaced at shallower depths in the upper crust (Plunder et al. 2022), typically as bodies up to 1 km in length and in swarms of 10–100 individual intrusions (Černý 1991).

A comprehensive exploration model for economic-grade lithium pegmatites must encompass a mineral system approach (Wyborn et al. 1994; Hagemann et al. 2016), which at its simplest defines a source, a migration pathway and a trap, as well as the preservation mechanisms that enable a deposit to persist. Much recent work has focused on the source of pegmatites (Černý 1991; Simmons et al. 1995; Shaw et al. 2016; Müller et al. 2017; London 2018; Koopmans et al. 2024) and preservation of their magmatic mineralogy, including the primary lithium ore minerals spodumene and petalite (Maneta et al. 2015; Ballouard et al. 2020; Wilde et al. 2021; Shaw et al. 2022; Pfister et al. 2023). However, the control that pathways such as faults and folds play on the distribution and size of economic pegmatites is relatively poorly understood (Hall and Kisters 2012; Silva et al. 2023; Gardiner et al. 2024).

Globally, pegmatites have a clear spatial relationship with regional structures such as faults and shear zones on scales up to 10 s of km (Kontak et al. 2005; Dill et al. 2012; Deveaud et al. 2013). Most importantly for exploration, the majority of large lithium pegmatite deposits are spatially associated with significant shear zones (Partington et al. 1995; Sweetapple 2000; Selway et al. 2005; Kremer 2010; Morissette et al. 2022). Further, detailed studies of pegmatite districts have shown how such structures may affect the distribution and remobilization of mineralized pegmatites within an individual district (Keyser et al. 2023; Silva et al. 2023), highlighting how regional stresses, and the resultant structures, affect the orientation, shape, and (re)crystallization of pegmatites. A key question relates to whether structures such as brittle faults and ductile shear zones play a role in pegmatitic melt migration and emplacement by providing a fundamental tapping mechanism, or do pegmatitic melts simply migrate passively through pre-existing crustal weaknesses (cf. Vanderhaeghe 1999; Brown et al. 2011; Hall and Kisters 2012)?

Here, we address this question through a comprehensive structural study of the Archean Zulu Pegmatite Field (Main pegmatite: 24.75 Mt at 0.43% Li_2_O, Premier African Minerals Limited 2024), situated within the Fort Rixon–Shangani greenstone belt in the south of the Zimbabwe Craton. The Zulu Pegmatite Field offers an excellent demonstration of how a combination of the structural history, hosting rocks, the spatial association with regional granitic bodies, major lithological variations, and crustal-scale fault systems affect the formation and distribution of mineralized lithium-bearing pegmatites. Using geological and structural mapping, field observations, and thin section microscopy, we demonstrate how the stress regime, major lithological competency contrasts, and structural history together influenced the localization and kinematics of shear zones and controlled the geometry, texture, mineralogy and degree of recrystallization of pegmatites during emplacement.

## Geological setting

### Geological history

**Fig. 1:**
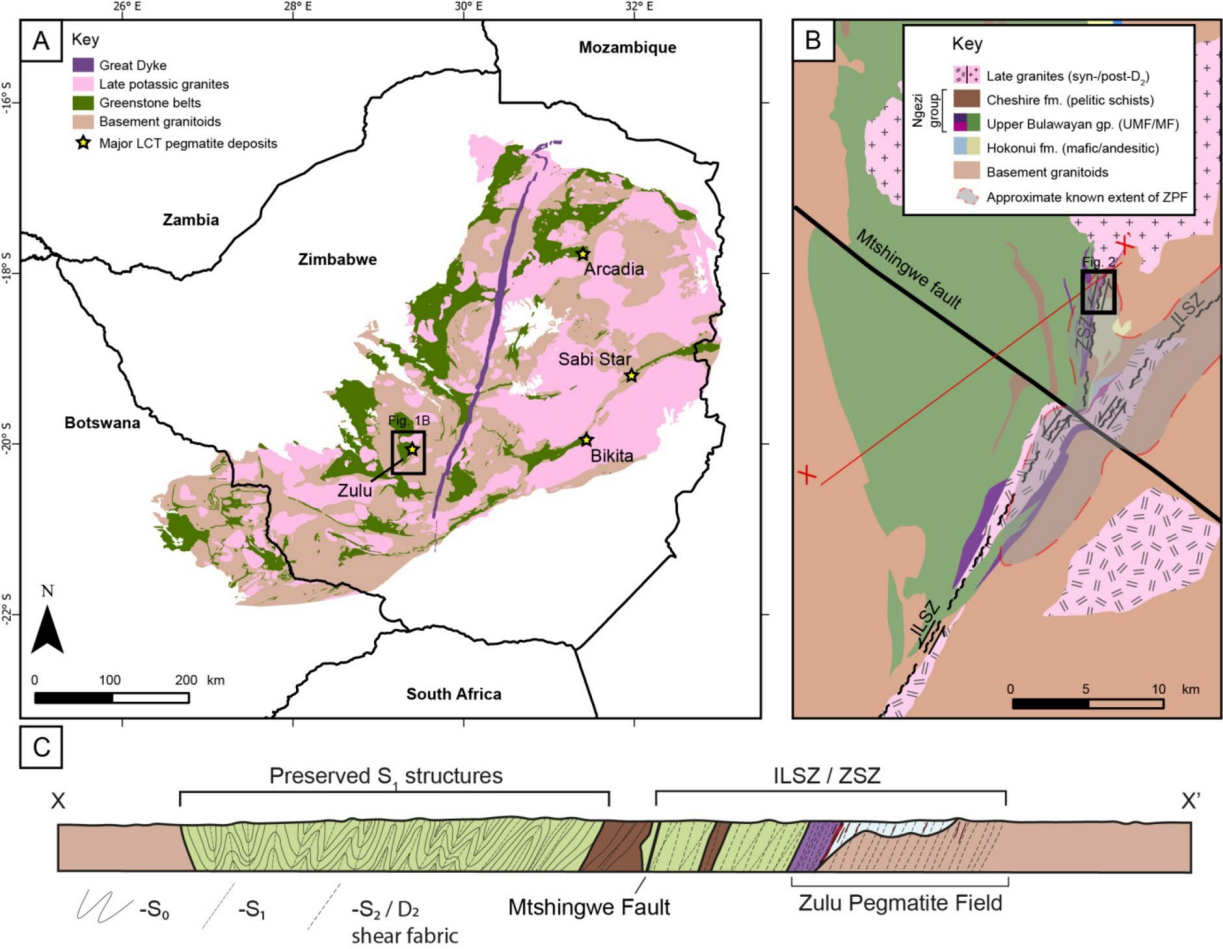
**A**) Simplified geological map of the Zimbabwe Craton, adapted from Ncube (1994). Major lithium deposits of Archean age are shown with yellow stars. Location of Fig. 1B noted by black square. **B**) Regional geological map around the core of the currently known Zulu pegmatite field, adapted from Harrison (1969). Location of Fig. 2 noted by black square. **C**) Schematic cross section X-X’ through the Fort Rixon – Shangani greenstone belt, highlighting structural fabrics preserved in the belt. ILSZ: Irisvale – Lancaster Shear Zone, ZSZ: Zulu Shear Zone, ZPF: Zulu Pegmatite Field

The Zulu Pegmatite Field is situated in the centre of the Zimbabwe Craton, a large cratonic block predominantly exposed in Zimbabwe (Fig. 1). The Zimbabwe Craton experienced two major magmatic-metamorphic events during the Archaean (Wilson et al. 1995), resulting in a series of folded granite-greenstone belts within a deformed granitoid gneiss terrane (Fig. 1). Early felsic crust formation occurred at c. 3600–3200 Ma, with magmatic activity preserved in the central Tokwe and Rhodesdale segments (Horstwood et al. 1999; Hofmann et al. 2022), located between the Zulu and Bikita pegmatite fields (Fig. 1). These segments formed the cratonic nuclei around which the rest of the craton accreted (Jelsma et al. 2021), with the majority of craton growth occurring during c. 3000–2500 Ma. Magmatism culminated during the Neoarchean with the emplacement of abundant potassic granites (the Chillimanzi and Razi suites; Wilson et al. 1995; Rollinson 2022; Chagondah et al. 2023). The formation of several lithium-bearing pegmatite fields (Zulu, Bikita and Arcadia) was contemporaneous with this late felsic magmatism, and a tentative genetic link has been placed between the pegmatite fields and neighbouring granites by some authors based on spatial relationships and geochronological overlap (e.g., Chagondah et al. 2024). Emplacement of the Great Dyke signifies final craton stabilization at c. 2575 Ma (Armstrong and Wilson 2000; Jelsma et al. 2021).

**Fig. 2:**
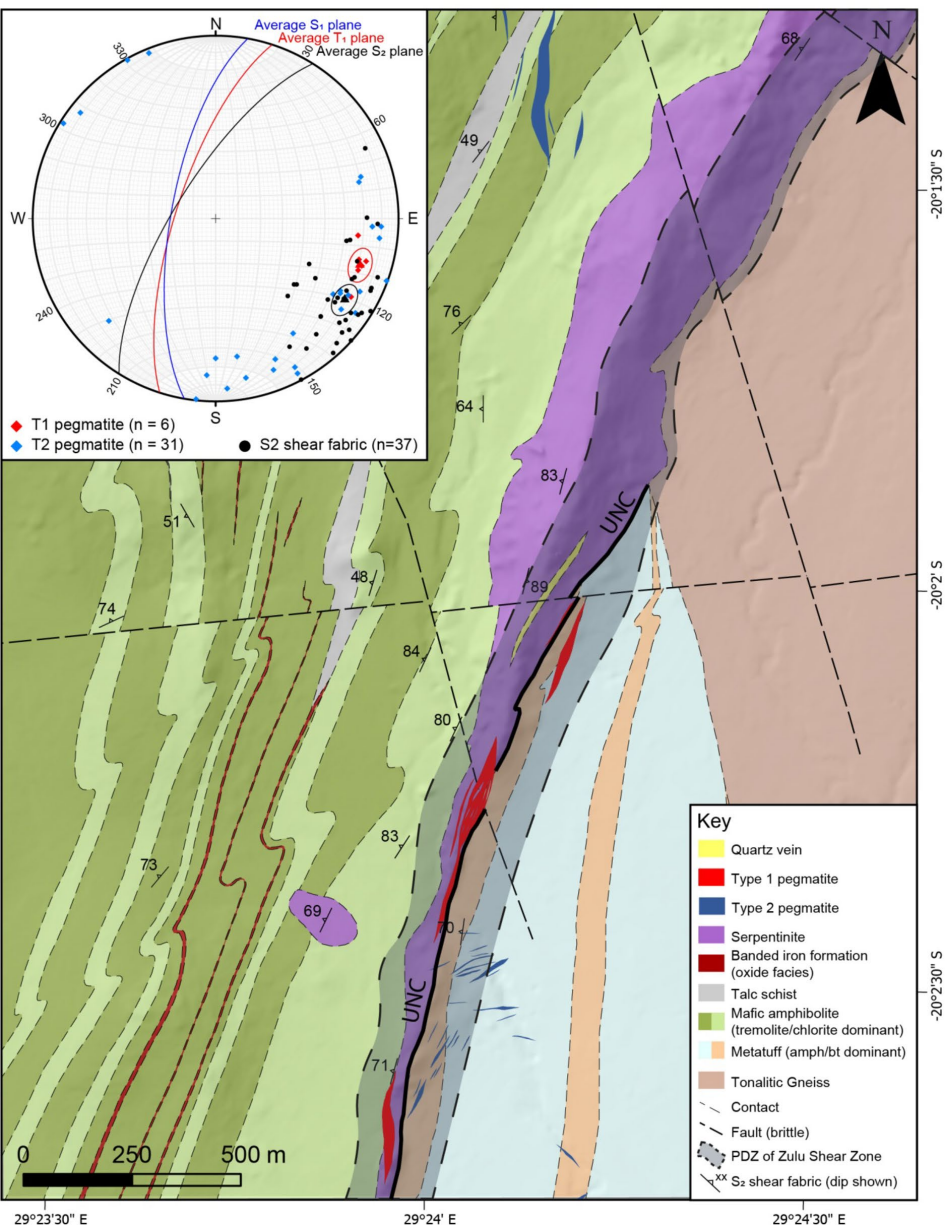
Inset: stereonet denoting main structural fabrics within the Zulu pegmatite field. A simplified description of each fabric can be found in Table 1. Main map: geological map of the region surrounding the major pegmatites of the Zulu pegmatite field. PDZ: principal deformation zone, UNC: major lithostratographic unconformity between the lower Hokonui and Ngezi formations

The c. 3000–2700 Ma Fort Rixon–Shangani greenstone belt (FRSGB) is situated 60 km north-east of Bulawayo (Fig. 1). It is exposed as a major syncline with a N-S axis and is dominantly comprised of mafic/ultramafic extrusive volcanic units (Harrison 1969). Large pegmatites of the Zulu Pegmatite Field were emplaced along the eastern margin of the Fort Rixon section of the greenstone belt, where a basal mafic/andesitic tuff succession is unconformably overlain by a mafic/ultramafic extrusive volcanic succession with minor sedimentary input (Figs. 1 and 2). These units can be respectively correlated to the Hokonui and Ngezi Formations (with associated intrusive rocks) of the Belingwe Greenstone Belt (Bickle et al. 1993) to the south-east (Prendergast 2004). Following deposition and emplacement, the greenstone belt underwent regional metamorphism to upper greenschist-facies conditions, which overprinted and recrystallized most of the primary magmatic fabrics (Harrison 1969). The age of this regional metamorphic episode is currently unknown.

**Table 1:** Summary of structural events in the Fort Rixon – Shangani greenstone belt

Phase of deformation	Description	Associated structures	Mean attitude of fabrics*
D_1_	• WNW-ESE directed shortening • Bedding parallel to F_1_ isoclinal folding • Weak axial planar S_1_ development	F_1_ S_1_	190/70 190/70
D_2_	• NNW-SSE directed shortening • S-shaped and Z-shaped F_2_ kink folds • Ductile sinistral shearing along ILSZ and associated 2nd order structures • Shear fabric (S_2_) development	F_2_ S_2_	68/167** 208/72
D_3_	• NW-SE directed shortening? • Brittle dextral faulting along WNW-ESE Mtshingwe fault system	Fault planes Dolerite-filled tension gashes	WNW – ESE NNE – SSW

*Attitude is presented as strike/dip using right-hand-rule conventions.

** Plunge and trend of fold axis

A series of potassic granites intruded into and around the greenstone belt; three smaller synkinematic porphyritic intrusions granites, a sheared porphyritic granite along the core of the Irisvale–Lancaster shear zone, and the postkinematic Nalatale granite, which almost completely transects the belt north of the Mtshingwe fault, (Fig. 2, Harrison 1969; Campbell and Pitfield 1994). These granites have been tentatively correlated with the c. 2650–2600 Ma Chilimanzi suite granites (Jelsma et al. 1996; Chagondah et al. 2023). There is no clear genetic relationship between the Zulu Pegmatite Field and any of the spatially associated granites.

### Structural history

The FRSGB experienced three phases of regional deformation (Table 1). The oldest phase (D_1_) consisted of folding by WNW-ESE directed shortening, which resulted in a major isoclinal F_1_ syncline, and a weak axial-planar S_1_ foliation (Campbell and Pitfield 1994).The second phase of deformation (D_2_) marked a change in shortening direction from WNW-ESE to NNW-SSE resulting in the formation of large, open, S-shaped warps and tight Z-shaped F_2_ kink folds, as well as the formation of a regional-scale transpressive sinistral wrench fault system concentrated along the major NE-SW Irisvale–Lancaster shear zone (ILSZ) and along major lithological boundaries such as the NNE-SSW trending Zulu Shear Zone (ZSZ) (Stowe 1980). This D_2_ event locally produced a penetrative shear fabric (S_2_) which locally transposed S_1_ fabrics into a parallel orientation. Both sinistral and later dextral reactivation shearing has been identified along the ILSZ (Campbell and Pitfield 1994).

The third phase of deformation (D_3_) consisted of NNW-SSE directed shorting, which formed the major NW-SE trending Mtshingwe Fault (Fig. 2, Harrison 1969). This fault system drags the earlier S_0_/S_1_ and D_2_ shear zones from striking NNE-SSW to NE-SW. The drag folds and the en-echelon dolerite dyke emplacement filling the fault-system suggest a dextral sense of movement with significant lateral displacement (Campbell and Pitfield 1994). The Mtshingwe Fault crosscuts the Great Dyke (c. 2575 Ma) further to the south-east.

## The Zulu pegmatite field

### The Zulu pegmatite field

The Zulu Pegmatite Field contains > 100 individual intrusions that lie along a generally NNE–SSW trend (Figs. 1 and 2). Pegmatites of the Zulu Pegmatite Field are LCT-type (after Černý et al. 2012), or Group 1 pegmatites (after Wise et al. 2022), and are significantly enriched in lithium (Harrison 1969; Goodenough et al. 2025). The largest bodies occur along the intrusive boundary between the Sonop serpentinite and the metamorphosed mafic/andesitic tuffs, although smaller intrusions occur up to 30 km towards the NE within the basement granitoids (Stowe 1968). The primary mineralogy of bodies in the Zulu Pegmatite Field typically consists of petalite (5–25%), quartz (25–30%), K-feldspar (10–30%), albite (5–30%), and muscovite (5–10%). Abundant spodumene (up to 30%) has also been described in the larger pegmatites, and also occurs within some of the smaller pegmatites hosted within the basement granitoids (Premier African Minerals Limited 2023).

Individual pegmatite bodies vary in width from 0.5 m to ~ 50 m, and some strike for up to 2 km. Contacts are sharp with the host rock. Two distinct pegmatite types can be distinguished based on mineralogy and texture (summarized in Table 2):

**Table 2:** Summary of (re)crystallization events in the Zulu pegmatite field

Event	Pegmatites affected	Description and primary mineralogy	Crystal Size	Li-aluminosilicate textures
1 – Magmatic	Type 1 + 2	• Spodumene (Spd 1, Type 1 only), Petalite (Type 2 only), K-feldspar, Quartz, Muscovite, Albite	5–15 cm	Classic SQUI (Type 1), Petalite (Type 2)
2 – Dynamic recrystallization	Type 1	• Albite, quartz, spodumene, muscovite (purple), ± spessartine • Strong fabric development, strong alignment of albite + muscovite, pre-/syn-kinematic spessartine growth	1–5 mm	Equant spodumene, fine grained
3 – Albitization	Type 1 + 2	• Albite, quartz, muscovite (green), ± spessartine • Weak/absent alignment of albite	1–5 mm	N/A
4 – Late-spodumene	Type 1 + 2	• Spodumene, quartz ± muscovite (green) • Along fractures and cleavage planes within petalite (Type 2)	< 1–5 mm	Equant spodumene (Type 1), SQS (Type 2)
5 – Low-T hydrothermal	Type 1 + 2	• Fine grained clay assemblages	< 1 mm	N/A

*SQUI* spodumene–quartz intergrowths

*SQS* spodumene–quartz symplectites (after Breasley et al. 2025)

#### Type 1 pegmatites

**Fig. 3:**
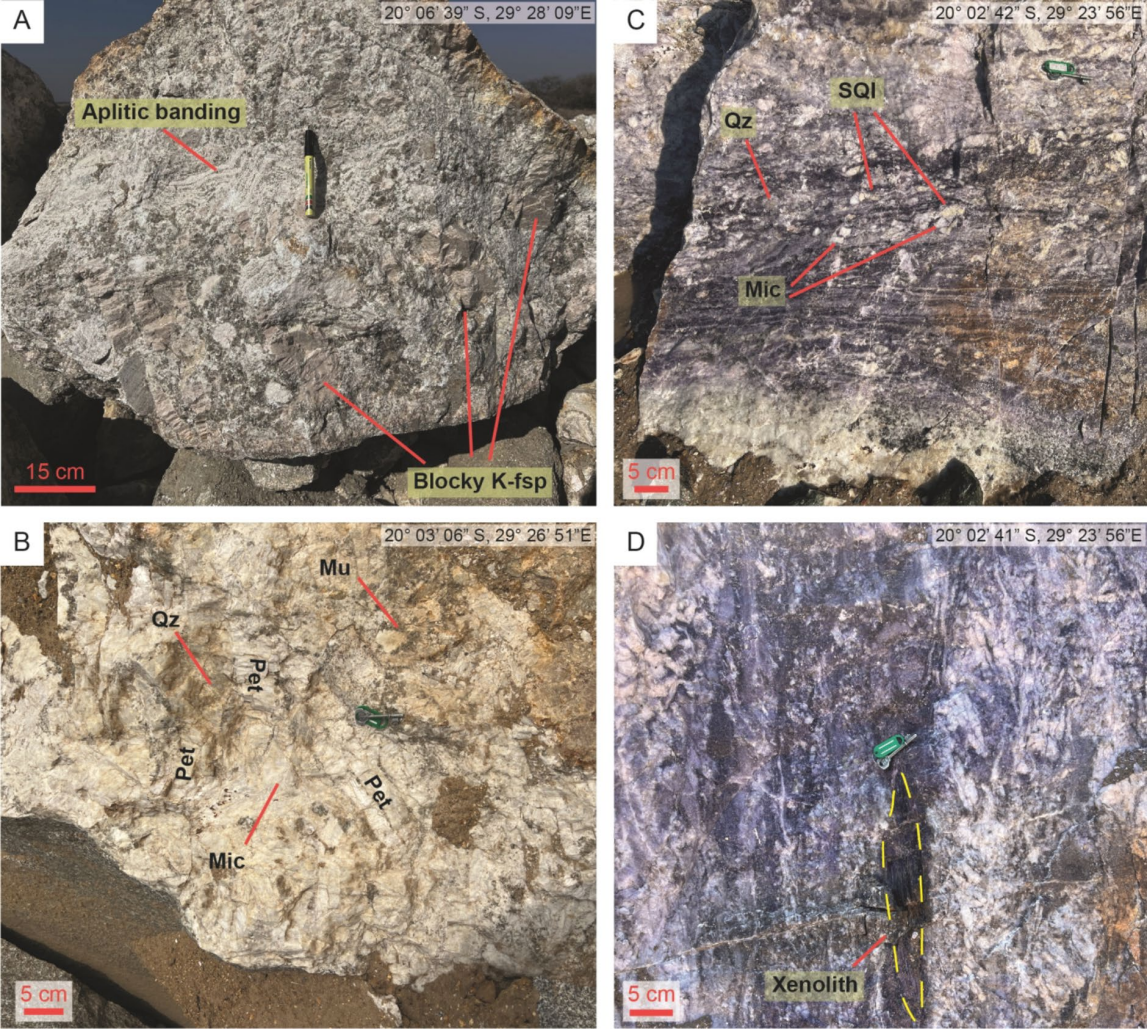
Representative photographs of pegmatites in the Zulu Pegmatite Field, with locations of images in top-right corner. **A**) Typical exposure of barren Type 2 pegmatite near the contact. Note the aplitic banding and unidirectional solidification texture expressed by the K-feldspar megacrysts. **B**) Mineralized Type 2 pegmatite with large petalite crystals randomly orientated in the intermediate zone. **C**) Typical exposure of the Type 1 pegmatites, with a strong fabric aligned left-right in the image. **D**) Rounded xenolith within Type 1 pegmatites within a localized high-strain domain. K-fsp: K-feldspar, Qz: quartz, SQI: spodumene-quartz intergrowths, Mic: microcline, Pet: petalite, Mu: muscovite

**Fig. 4:**
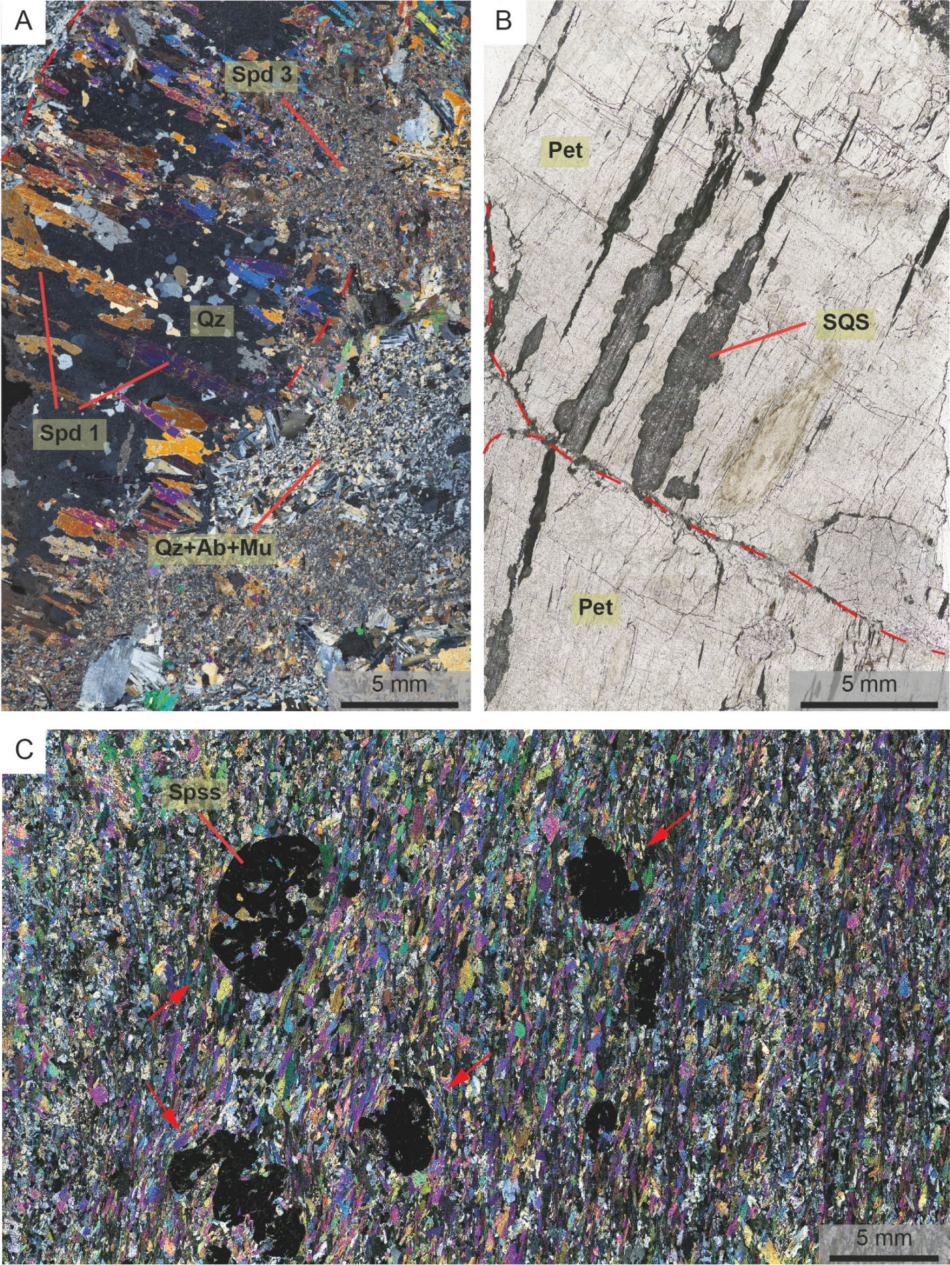
Photomicrographs from pegmatites in the Zulu pegmatite field. **A**) An example of Classic SQUI (Spd 1) within Type 1 pegmatites. Note the overprinting albitization (Event 3, Table 2) and subsequent late spodumene (Spd 3; Event 4, Table 2) events. **B**) Large petalite crystals in Type 2 pegmatites, with spodumene-quartz symplectites (SQS, Event 4, Table 2) occurring along petalite cleavage planes. **C**) A strongly foliated zone of a Type 1 pegmatite. Evidence of fabric-wrapping textures around spessartine (opaque mineral) highlighted in red arrows. Qz: quartz, Spd: spodumene, Ab: albite, Mu: muscovite, Pet: petalite, Spss: spessartine

Type 1 pegmatites attain widths up to 50 m and are therefore the volumetrically dominant pegmatite type in the field. Individual intrusions have asymmetric lenticular shapes with long axes subparallel to the principal deformation zone of their host shear zone (Fig. 2). The primary magmatic mineralogy is comprised of spodumene, K-feldspar, albite, quartz, and muscovite. Individual crystals are up to 15 cm long (Fig. 3C and D). An early generation of spodumene (Event 1, Table 2) in Type 1 pegmatites predominantly consists of crystals (0.1–1 cm) with a strong crystallographic orientation within oikocrystic quartz (Classic SQUI of Breasley et al. 2025; Figs. 3C and 4A).

**Fig. 5:**
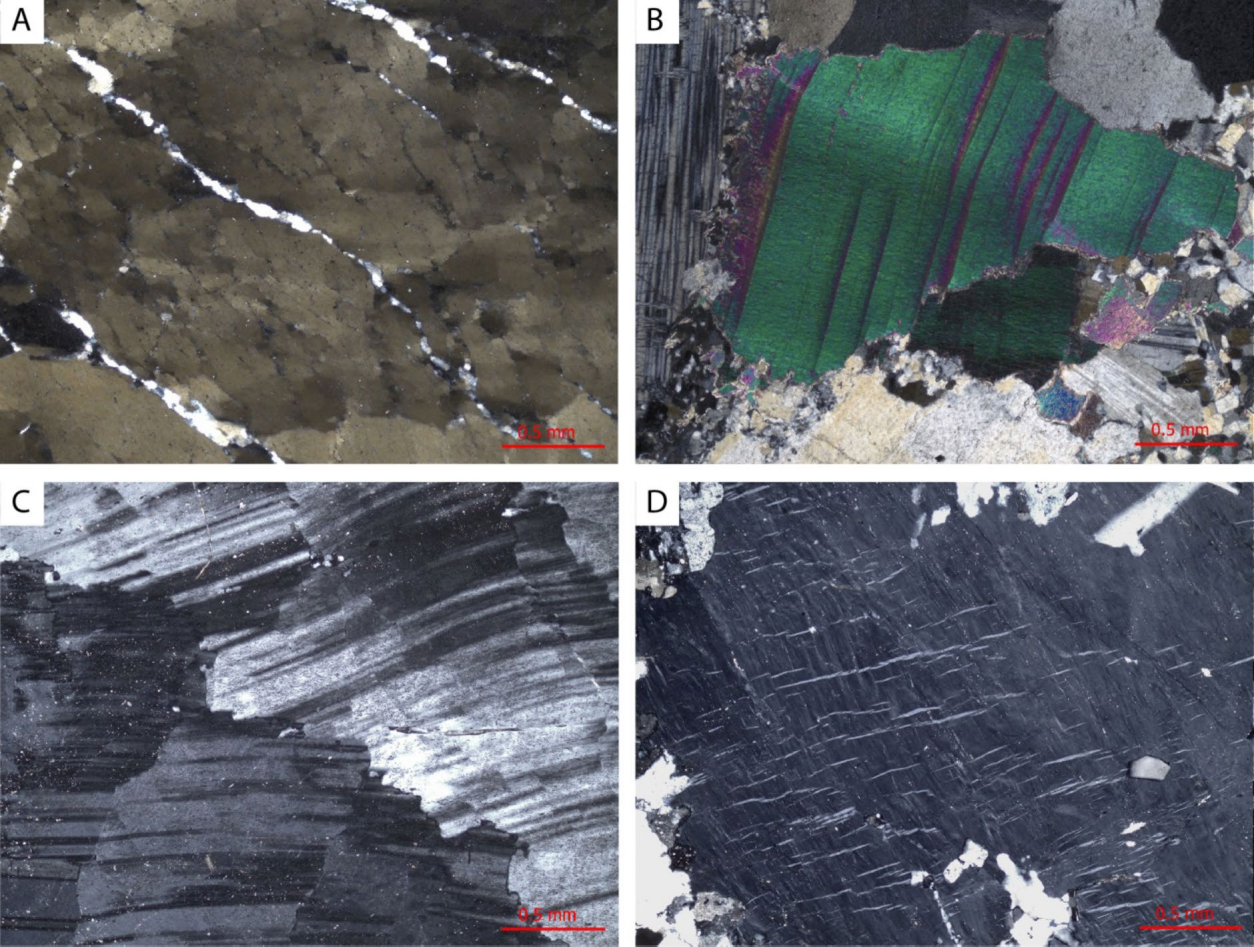
Typical deformation microstructures within Type 1 pegmatites. **A**) Chessboard extinction in relict magmatic quartz. **B**) Kinked cleavages within a relict muscovite crystal. **C**) Plastic deformation of early albite twins. **D**) Orientated flame perthite within early microcline

Primary textures are otherwise poorly preserved. Where magmatic mineralogy is preserved, quartz crystals commonly exhibit chessboard and undulose extinction patterns, whereas plastic deformation of lamellar twins in coarser albite is common (Fig. 5A and C). Microcline has well-developed tartan twinning and flame perthite is prevalent (Fig. 5D). Primary muscovite, where preserved also exhibit kinked cleavage planes (Fig. 5B).

**Fig. 6:**
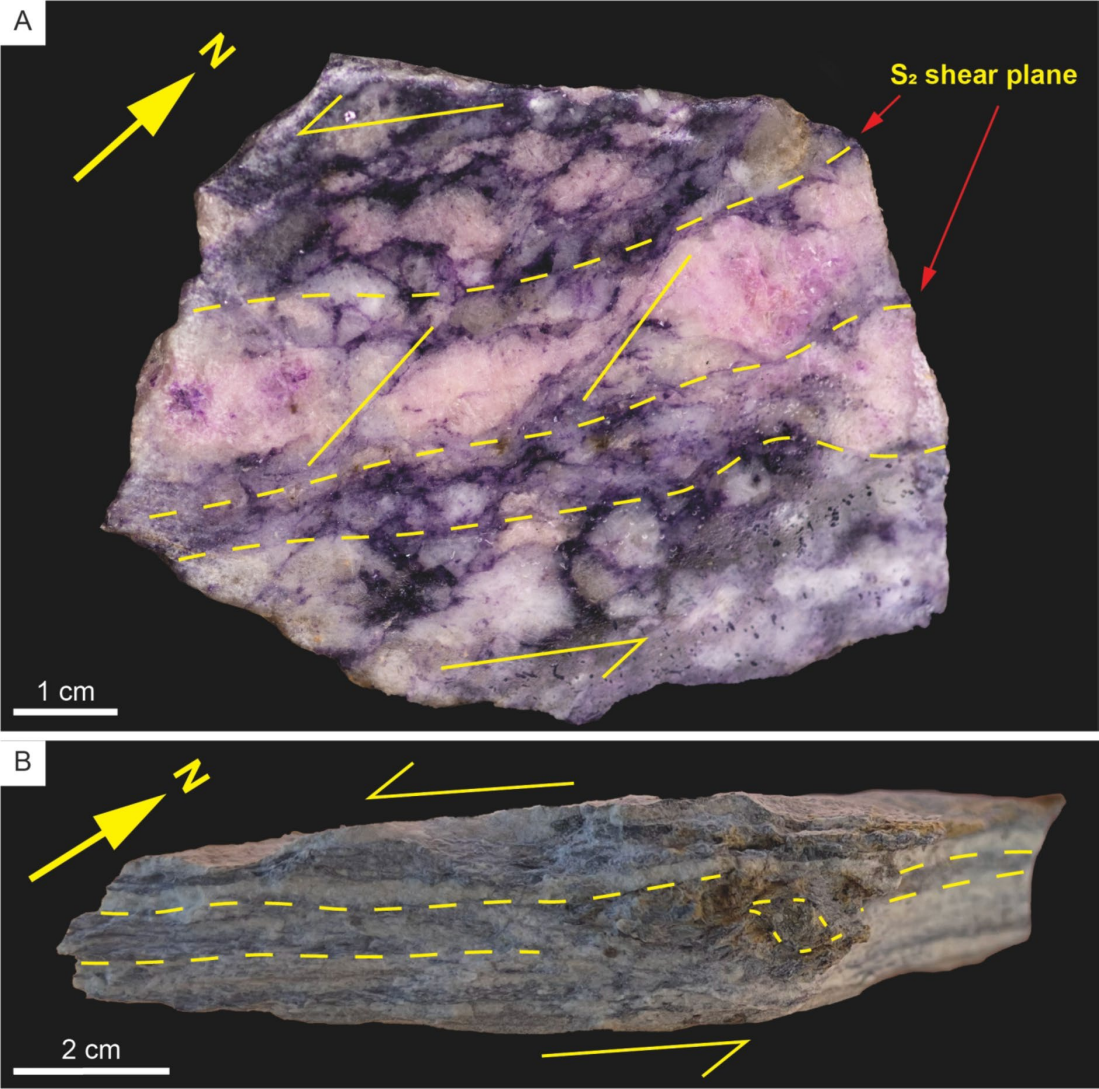
Hand samples of Type 1 pegmatites. **A**) Sheared pegmatite with fabric defined by aligned mica sheets, and antithetic rotation within relict SQUI grains. **B**) Protomylonitic pegmatite with bands defined by albite and lithian muscovite. Rotated spessartine porphyroblasts indicate the sense and direction of shear

An early deformation-driven recrystallization event (Event 2, Table 2) is pervasive and largely replaced the primary mineralogy with a fine-grained (1–5 mm) albite + quartz + spodumene + muscovite ± spessartine assemblage (e.g., Fig. 3C). Relict magmatic crystals are entrained as porphyroclasts (retaining deformation microstructures described above), and the muscovite has a deep purple colour in outcrop (Figs. 3C and D and 6A). Individual crystals of spodumene within the replacement mineralogy are equant, although albite and muscovite within these aggregates are commonly strongly aligned to define a fabric within the pegmatites (described in more detail below). Locally, this unit developed a strong schistosity (possibly owing to a horizon of less competent magmatic mineralogy within the pegmatite), and euhedral spessartine is interpreted to be pre- to syn-kinematic with respect to the fabric (Figs. 4C and 6B).

Later albitization (Event 3, Table 2) is most common along the hanging wall of the pegmatites, producing zones between 5 cm and 2 m wide of albite + quartz + muscovite and rare spessartine with a weak fabric (Fig. 4A). Sporadic pockets of albitization also occur internal to the pegmatites. The muscovite in the albitized zones is typically light-green/grey in color. A later, spodumene-rich, replacement unit (Event 4, Table 2) is comprised of fine grained (< 1 mm) spodumene + quartz + albite + muscovite with no defined orientation. This unit is only locally present and can be seen to overprint all previous crystallization events in the pegmatite (Fig. 4A). Subsequent low temperature hydrothermal alteration products (Event 5, Table 2) are uncommon, though locally spodumene and feldspars are replaced by fine grained clay minerals.

#### Type 2 pegmatites

These are characterized by their relatively simple, magmatic mineralogy and textures. Individual intrusions are often limited in width, being no greater than 5 m. A simple pegmatite zonation is common, with a fine-grained border zone (10–30 cm) followed by a zone of irregular aplitic banding (5–30 cm) defined by alternating muscovite-rich and muscovite-poor bands subparallel to the contact (Fig. 3A). A coarse-grained intermediate zone and a poorly developed quartz core occur towards the centre of the pegmatite bodies (Event 1, Table 2). The intermediate zone consists of crystals up to 15 cm in size and can be further subdivided into a mineralized (Fig. 3B) and barren (Fig. 3A) subgroup. Petalite (in the mineralized subtype), blocky K-feldspar (in the barren subtype), quartz, albite, and muscovite constitute the major minerals in the intermediate zone. Crystals are commonly euhedral to subhedral. Fabric-forming recrystallization (Event 2, Table 2) is not observed within the Type 2 pegmatites.

Locally Type 2 pegmatites have been albitized (Event 3, Table 2), leading to replacement of the primary mineralogy by a fine-grained albite + quartz + muscovite assemblage. A muscovite + quartz greisen, predominantly along the margins of blocky K-feldspar and petalite, is sporadically developed. Some petalite in Type 2 pegmatites is partially replaced (Event 4, Table 2) by a complex symplectic intergrowth of spodumene + quartz along cleavage planes and fracture faces (Fig. 4B). Evidence for further low-temperature alteration (Event 5, Table 2) is sparse, although feldspars and petalite are patchily replaced with clay-type minerals.

#### Lithostructural observations on the Zulu pegmatite field

Competency contrasts between lithologies generated distinct high-strain and low-strain domains during D2 deformation. Across the study area the predominant foliation dips steeply towards the WNW (averaging 212° strike/72° dip) across a region > 5 km wide.

**Fig. 7:**
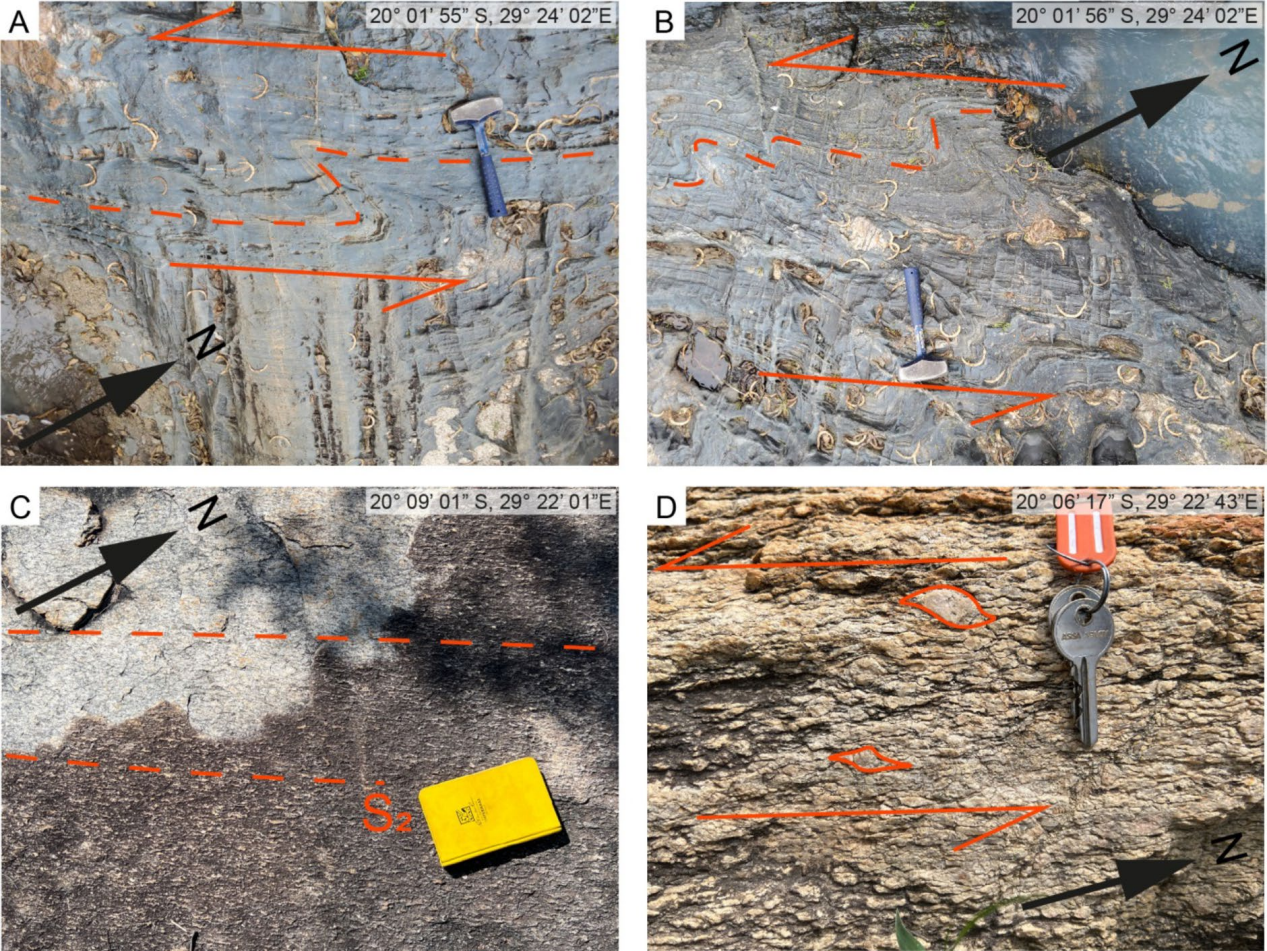
Top-down outcrop images of host rocks within the Zulu Pegmatite Field. Location of images in top right. **A**, **B**) S-shaped kink folds indicating sinistral sense of shear within mafic/ultramafic extrusives. **C**) S_2_ fabric preserved in moderately deformed basement granitoids highlighted by the alignment of feldspar crystals. **D**) sigma-type clasts within highly deformed basement granitoids

Within the basement granitoids the S_2_ fabric is defined by the alignment of biotite and feldspar (Fig. 7C, D). Strain appears to be heterogeneously distributed through the granitoids, with weakly foliated granitoids gradually obtaining strongly deformed and banded fabrics on the scale of 10 s of m (Fig. 7C). Local megacrystic granitoids within higher strain domains preserve sigma-type clasts indicating a sinistral, SSW oriented, sense of shear (Fig. 7D).

**Fig. 8:**
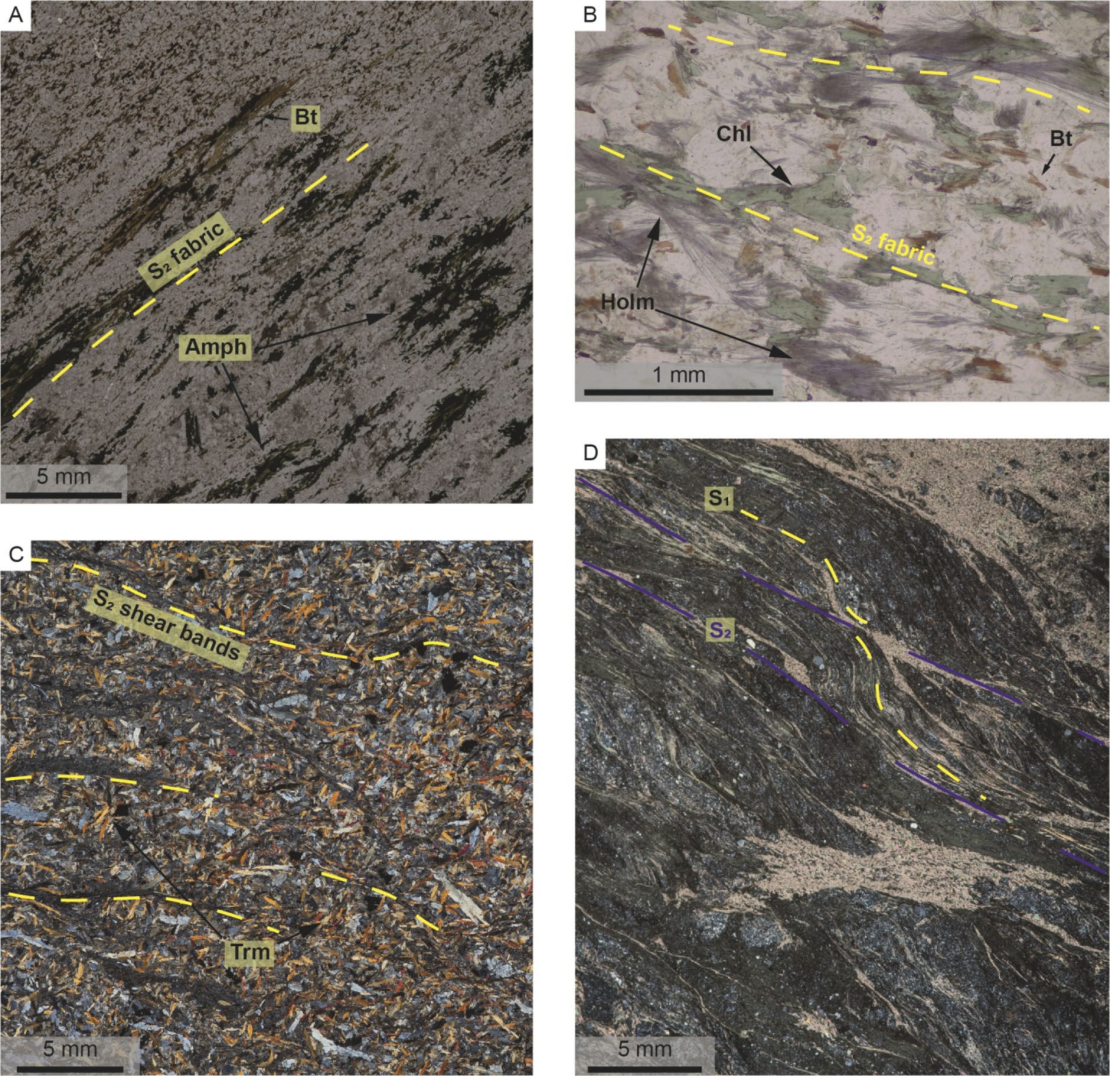
Thin section images of host rocks in the Zulu Pegmatite Field. **A**) Protomylonitic andesitic tuff with the fabric defined by highly attenuated biotite and amphibole crystals. **B**) Strongly altered andesitic tuff adjacent to a Type 1 pegmatite. Alteration phases are all strongly aligned to S_2_. **C**) S_2_ chlorite shear bands within a mafic/ultramafic extrusive in a low strain domain. **D**) Highly sheared serpentinite highlighting the relationship between the earlier S_1_ fabric (yellow dashed lines) with the well-developed shear fabric (S_2_, blue lines) forming S/C fabrics within the principal deformation zone of the Zulu shear zone. Bt: biotite, Amph: amphibole, Chl: chlorite, Holm: holmquistite, Trm: tremolite

The basal mafic/andesitic tuff succession on the eastern margin of the FRSGB is locally protomylonitic with the S_2_ fabric defined by the alignment of relict amphibole and biotite crystals (Fig. 8A). Similar mylonitic fabrics are preserved in the Sonop serpentinite, where S/C fabrics and relict phenocrysts also indicate sinistral shearing (Fig. 8D). Low-strain domains are rarely observed within the Sonop serpentinite. S_2_ high strain domains within the overlying mafic/ultramafic succession are defined by the alignment of tremolite/actinolite and chlorite-rich shear bands (Fig. 8C). Low-strain domains occasionally preserve a S_1_ fabric that is locally overprinted by thin, chlorite-rich shear bands (Fig. 8C). S-shaped kink folds are common within the mafic/ultramafic succession and suggest sinistral NNE-SSW orientated shearing (Figs. 7A, B).

**Fig. 9:**
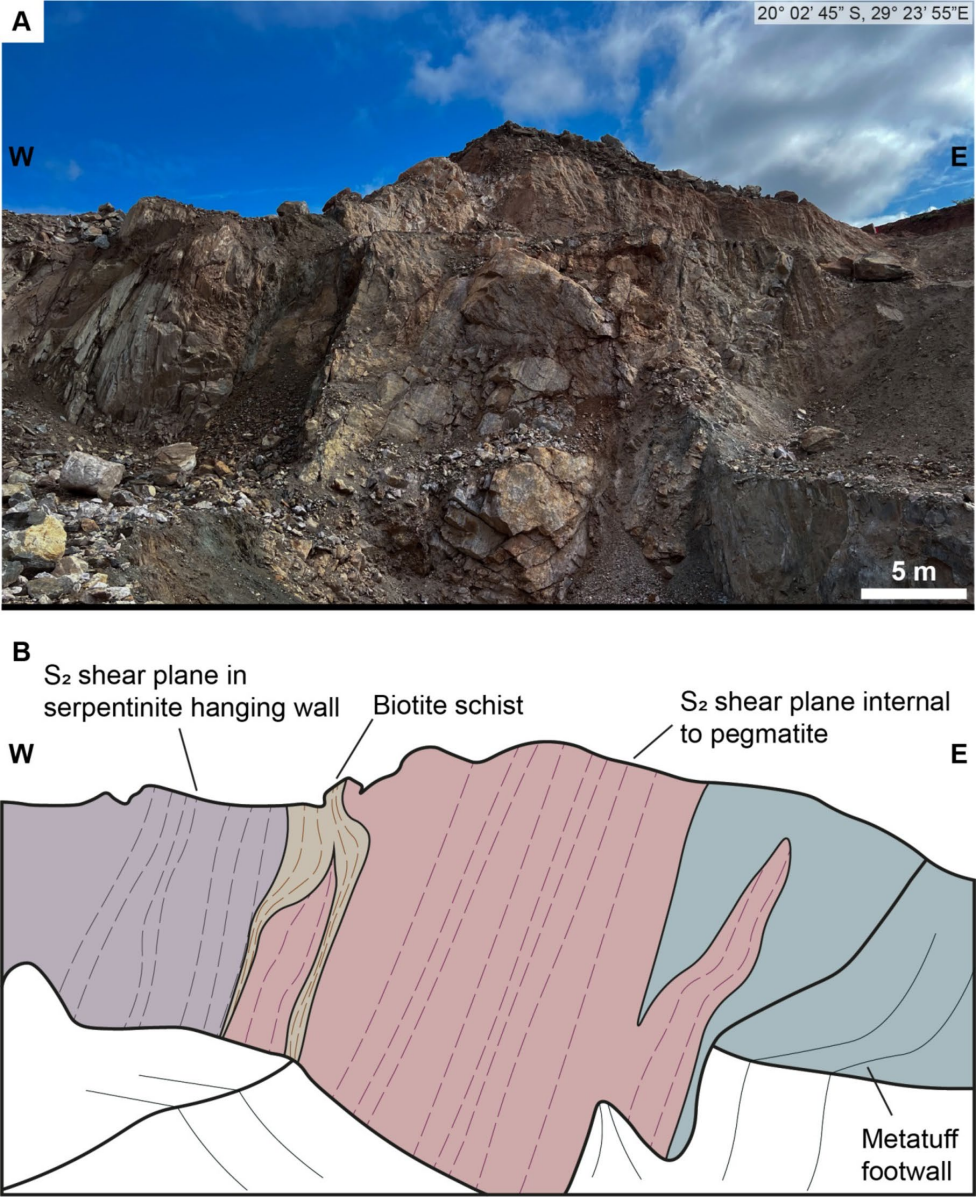
**A**) Outcrop image of a Type 1 pegmatite in an excavated pit. Image traced in B. **B**) Relationship of the S2 fabric within the host rocks and the fabric internal to the pegmatite highlighted, with the common biotite schist along the hanging wall of the pegmatite. Location of image in top right

Type 1 pegmatites are primarily emplaced within high-strain domains along the contact between the Sonop serpentinite and underlying mafic/andesitic tuff successions. Host rocks to Type 1 pegmatites have been affected by exomorphic wall rock alteration adjacent to the pegmatites which largely destroyed their pre-intrusion mineralogy. A biotite + quartz alteration zone, up to 20 cm thick, is typically observed along the serpentinite hanging wall with a pervasive foliation subparallel to S_2_ (Fig. 9). Asymmetric crenulations are common within the biotite-rich domains. Along the footwall of the pegmatites, a chlorite + biotite ± holmquistite alteration halo within the mafic/andesitic tuff succession is commonly observed, with the acicular amphibole and platy mica showing a preferred alignment subparallel to S_2_ (Fig. 8B). Fabrics defined by the alignment of albite and mica in the early dynamically recrystallized zones, together with the schistosity preserved in high-strain domains, are all subparallel to the long axis of the pegmatites and S_2_ (Fig. 9).

**Fig. 10:**
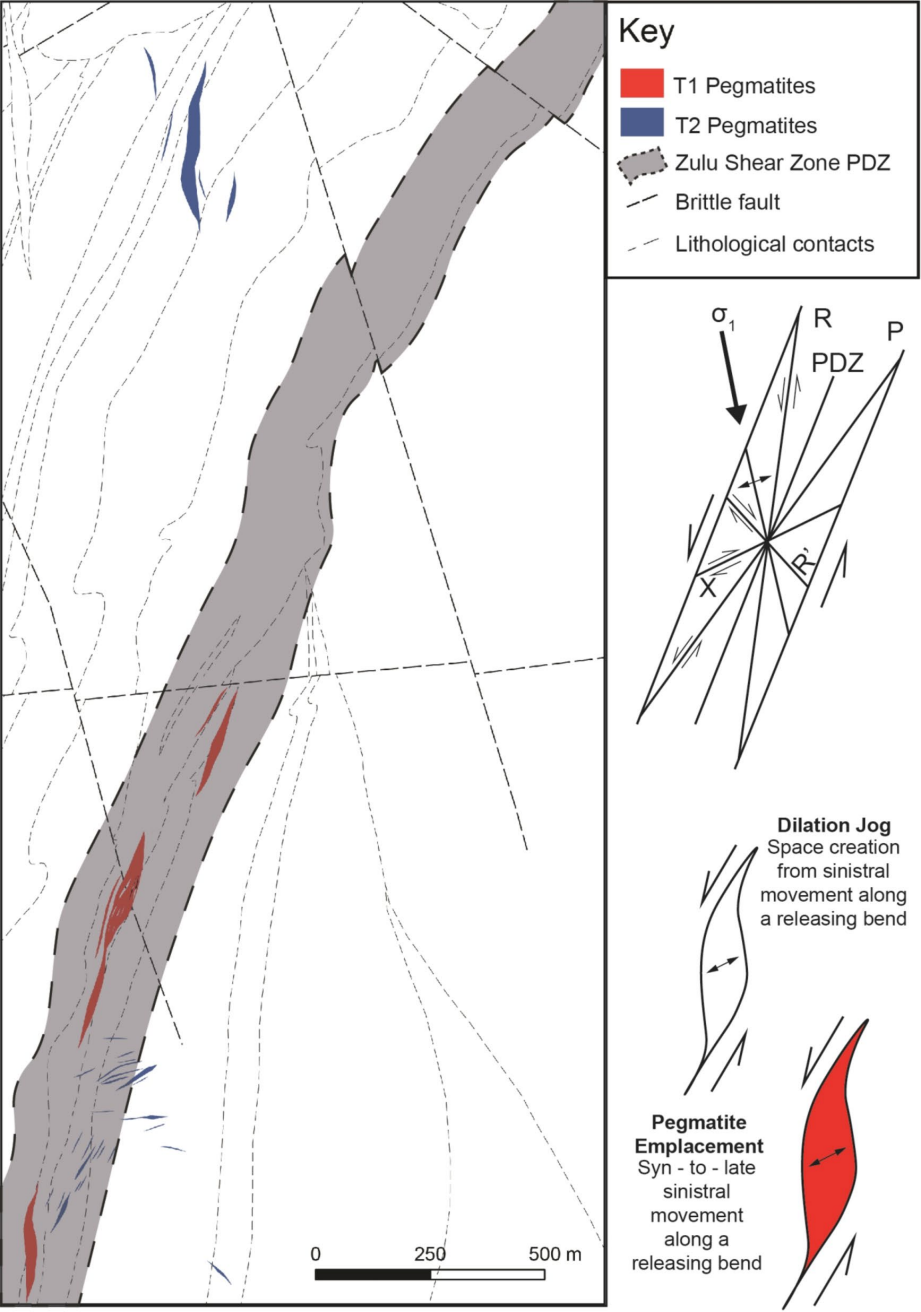
Simplified geological map discriminating between group one and two pegmatites. A schematic emplacement model of the Zulu Pegmatite Field with a parallel Riedel schematic is drawn to the right of the map. T1: Type 1, T2: Type 2, PDZ: principal deformation zone

Type 2 pegmatites are mostly emplaced within the basement granitoids and low-strain domains within the basal mafic/andesitic tuff successions, and often obliquely (30–90°) crosscut the primary fabric (Fig. 10). There are no apparent deformation fabrics within Type 2 pegmatites, and only minor alteration can be observed within the wall rock.

Deformation features related to motion along the Mtshingwe fault (D_3_) are absent in regions containing Type 1 pegmatites. Type 2 pegmatites adjacent to D_3_ structures are only observed within the granitoid basement and are oriented oblique to the en-echelon dolerite dyke swarm within the Mtshingwe fault zone. In these examples, the primary magmatic mineralogy is pervasively altered to clays.

## Discussion

### Relative timing and structural setting of pegmatite emplacement at Zulu

Transcurrent sinistral shearing along the ISLZ and ZSZ defined by asymmetric dragging of the pre-existing S_1_ foliation along the S_2_ shear fabric, asymmetrical sigma clasts, and s-shaped kink folds developed along the margins of the high-strain zones (Fig. 7).

**Fig. 11:**
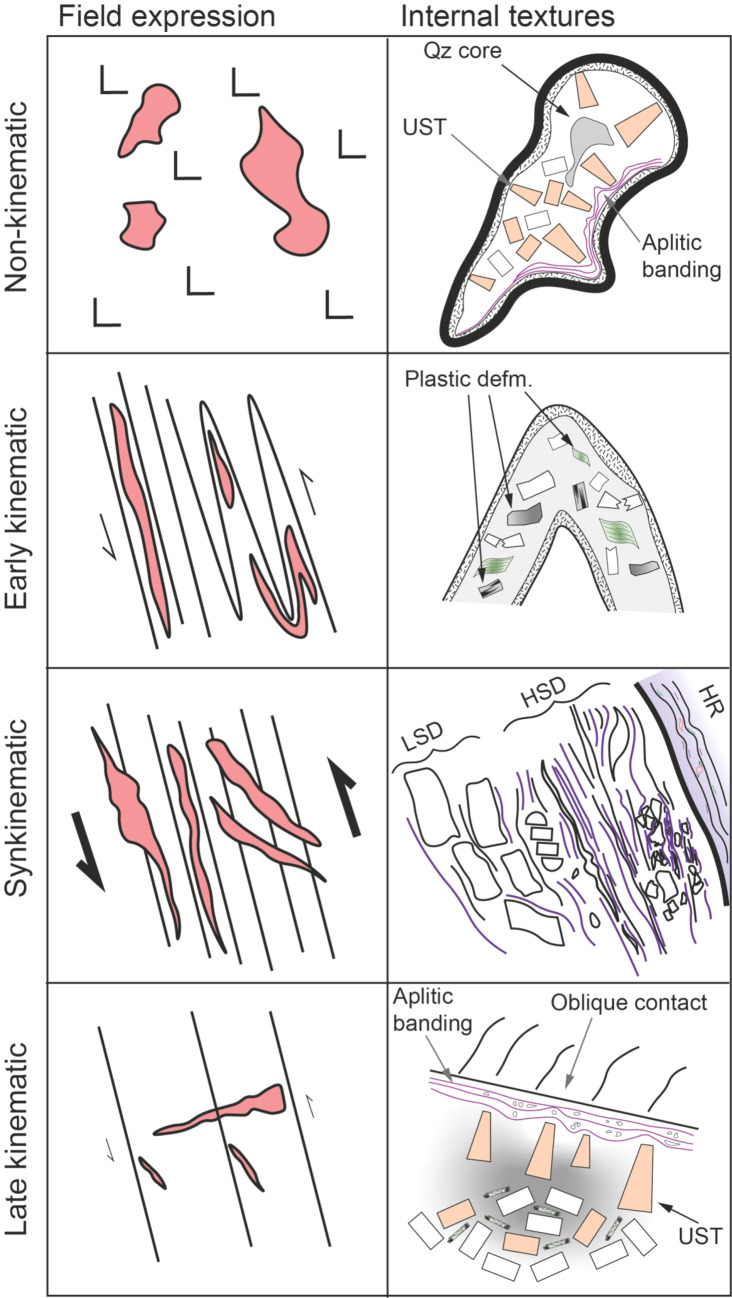
Schematic diagram highlighting the different styles of emplacement relative to the regional structure for pegmatites emplaced at different times during shear-dominated deformation. Both field expressions (left) and internal textures (right) are drawn. Non-kinematic pegmatites have no relationship to any fabric, nor preserve any dynamic recrystallization textures related to syn-emplacement deformation. Early kinematic pegmatites are likely to become tightly infolded with the shear fabric, and any magmatic phases preserved will exhibit extensive crystal-plastic deformation. In the syn-kinematic case, the fabric internal to the pegmatite is parallel, whilst the orientation of the intrusion locally crosscuts, deformation fabrics in the host rock. Syn-kinematic growth of exomorphic alteration phases provides additional evidence to support syn-kinematic emplacement, as is observed in Type 1 pegmatites in the Zulu pegmatite field. Late kinematic pegmatites will be emplaced into tension gashes and/or mode-1 fractures in the shear zone, and preserve a largely primary mineralogy, as is observed in Type 2 pegmatites in the Zulu pegmatite field. UST: unidirectional solidification textures, HSD: high-strain domain, LSD: low-strain domain, HR: host rock

Type 1 pegmatites are interpreted to have been emplaced syn- D_2_ shearing. Deformation within the pegmatites was accompanied by extensive recrystallization and remobilization of lithium, leading to the formation of lithian muscovite and the replacement of pre-existing lithium silicates with a later fine-grained generation of spodumene (Table 2). Critically, the syn-kinematic emplacement during sinistral shearing of the pegmatites is supported by three main lines of evidence: 1) their lenticular shape of some Type 1 pegmatites in agreement with emplacement into dilational jogs (in some cases, Type 1 pegmatites present a more contorted shape which may suggest deformation by subsequent shearing), because of which the orientation of the pegmatite-wallrock contacts locally cross-cuts the S_2_ fabric (Figs. 2 and 10). The internal fabric subparallel to S_2_ within pegmatites (Figs. 3, 5 and 8), and 3) the synkinematic growth of minerals within the exomorphic haloes surrounding Type 1 pegmatites, which indicates that country rock metasomatism must have occurred during active deformation (Figs. 8B and 11). The significant rheological contrast between the weaker, more ductile Sonop serpentinite and the more competent underlying metamorphosed tuffs (Fig. 2) likely acted as a concentrating mechanism, localizing melt flow during ascent (Papeschi et al. 2022).

In contrast to Type 1, the Type 2 pegmatites preserve a largely magmatic mineralogy, and have no internal fabric. Cross-cutting relationships with their host foliated granites indicate that these pegmatites were emplaced after the regional fabric was developed. Given the systematic orientation of individual bodies, we suggest that Type 2 pegmatites were syn-to-late-kinematic to D_2_ and emplaced along typical sinistral strike-slip subordinate fracture sets and/or tension gashes (Figs. 10 and 11). This timing, and emplacement into zones of lower strain, served to truncate the cooling history of Type 2, inhibiting extensive recrystallization and preserving primary magmatic mineralogy and textures.

As well as controlling the emplacement trajectory, host rock lithology also influenced the size of individual pegmatite bodies. As the Zulu pegmatite field extends away from the Fort – Rixon Shangani Greenstone belt into the basement granitoids, individual pegmatites become narrower in width. We interpret this to be driven by the absence of significant rheological contrast within the more competent granitoid host rocks. Whereas the relatively incompetent lithologies and lithological boundaries within the greenstone belt accommodated strain by ductile deformation and concentrated fluid flow along lithological boundaries, the more competent granitoid basement likely accommodated strain through localized brittle fracturing. Narrow mode-1 fractures formed as a result, facilitating pegmatite emplacement within the basement granitoids (Fig. 11). This behavior would have limited dilation, promoting the formation of several thin, strike-parallel intrusions, instead of thick bodies.

### Comparison with other pegmatite fields

Systematic structural analyses of economic pegmatite fields are rare in the literature. However, similar relationships to those observed in the Zulu pegmatite field have been described in other economic pegmatite districts and highlight the importance of regional-scale controlling structures on the size, orientation, and distributions of pegmatites themselves. Four well-studied examples are briefly mentioned here.

**Fig. 12:**
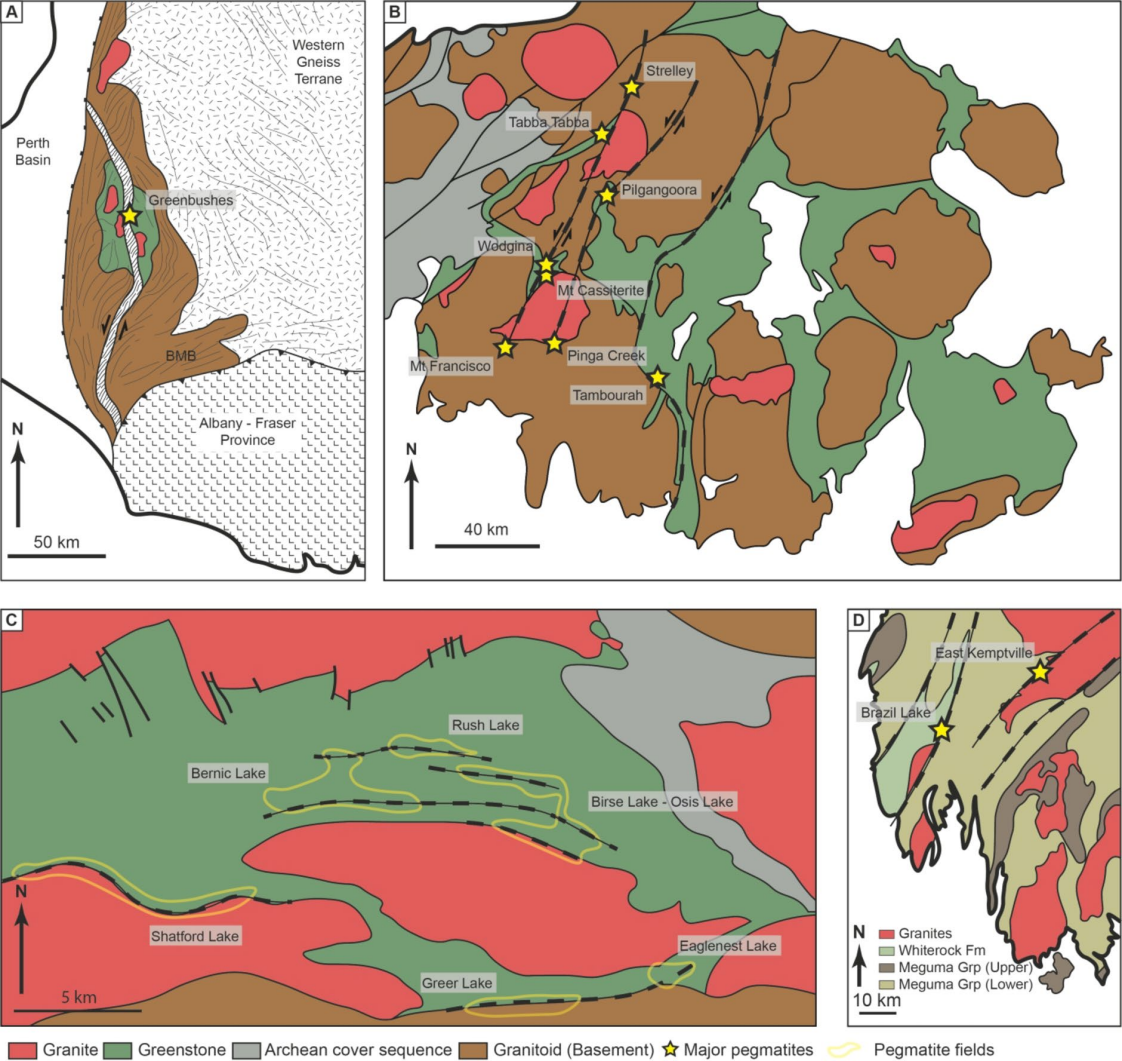
Example case studies from the **A**) Yilgarn Craton (after Partington et al. 1995), **B**) North-east Pilbara craton (after Sweetapple and Collins 2002), **C**) Winniper River pegmatite district (after Kremer 2010), and **D**) southwestern Nova Scotia (after Kontak 2006)

The Archaean Greenbushes pegmatite field, Western Australia, is one of the largest hard-rock Li deposits in the world (Bowell et al. 2020; Fig. 12A). The Greenbushes pegmatite is emplaced into the 150 km long and 15–20 km wide Donnybrook–Bridgetown shear zone, which preserves a major sinistral component of movement (Partington et al. 1986; Partington 1988). Detailed fieldwork has shown that mineralized pegmatites of the Greenbushes pegmatite contain relict igneous structures and pegmatitic microstructures indicating syn-kinematic emplacement into structures related to the Donnybrook–Bridgetown shear zone. Individual pegmatite bodies strike subparallel to their controlling structures, as with the Type 1 pegmatites in the Zulu pegmatite field.

Pegmatites of the north-eastern Archaean Pilbara craton in Australia are also associated with major regional structures (Fig. 12B). Several N–S to NNE–SSW trending sinistral shear zones transect the craton. Most large Li-rich pegmatites (e.g., Wodgina, Tabba Tabba) in the Pilbara are hosted within subparallel dilational sites where greenstone belts are cross-cut by these shear zones (Sweetapple 2000; Sweetapple and Collins 2002). The Zulu pegmatite field is similarly located at the transect between a major regional structure and a greenstone belt.

The Brazil Lake pegmatite field was emplaced into the Meguma terrane (540–420 Ma) metasedimentary and metavolcanic successions (Fig. 12D; Culshaw and Reynolds 1997; Kontak 2006). Shear zones are common in the area and were repeatedly reactivated during both deformational events. Pegmatites within the Brazil Lake pegmatite field were emplaced at 395 Ma along major lithological boundaries into dilational sites that have orientations sub-parallel to dextral strike-slip movement (Kontak 2006). Similarly to the Type 1 pegmatites of the Zulu pegmatite field, deformation along the pegmatite–wall rock contact and deformation within primary magmatic phases at Brazil Lake indicate that pegmatite emplacement was syn-kinematic, with shearing having continued post-crystallization.

The Winnipeg River pegmatite district is largely hosted within the Archaean Bird River greenstone belt and hosts several pegmatite fields, including the world-class Tanco deposit (Fig. 12C, Černý 1982; Gilbert et al. 2008). Three major deformation events have been recognized in the region and are summarized by Duguet et al. (2009). Most pegmatites within the Winnipeg River pegmatite district are spatially and temporally associated with reactivated D_3_ shear zones, such as the North Bernic Lake shear zone (Baadsgard and Černý 1993; Kremer 2010). Both ductile and brittle fabrics formed in response to D_3_, and pegmatites were emplaced into both ductile and brittle dilational sites which in some cases result in pegmatite orientations oblique to the main shear zone (Brisbin and Trueman 1982). As opposed to the Zulu pegmatite field, where the largest pegmatites formed syn-kinematic to ductile deformation, the largest pegmatites in the Winnipeg River pegmatite district (e.g., the Tanco pegmatite) appear to have occurred late-kinematic during colder, brittle deformation (akin to Type 2 pegmatites at in the Zulu Pegmatite Field, Kremer 2010). In this way the timing of peak melt flux may be an important factor in determining which structural setting within shear zones are most amenable to large pegmatite deposits.

These examples, together with our observations at Zulu, highlight that major pegmatite fields hosting economic deposits formed by syn-kinematic pegmatite emplacement along major ductile shear zones.

### The influence of shear zones on pegmatite migration and emplacement

In the mineral systems framework, understanding the formation, migration, and crystallization of pegmatitic melt from source to trap is critical to constrain the expected distribution of such ore bodies in a particular district at the current level of exposure (Wyborn et al. 1994; Hagemann et al. 2016; Gardiner et al. 2024). Whilst our observations of the Zulu pegmatite field are primarily focused on the trap (given the source is not exposed), the close association between the ILSZ and the Zulu Pegmatite Field inevitably leads to speculation on how such shear zones might be drivers and/or enablers of the localization and migration of pegmatitic melt, leading to the crystallization of economic pegmatites. This is irrespective of the source model (granite fractionation vs. anatectic) invoked, since regardless of melting mechanism, to form a large deposit significant volumes of pegmatitic melt must be extracted and physically accumulated within the mid-upper crust. Although pegmatitic melt migration into pre-existing structures may generate a spatial correlation without having to call upon a genetic association (Deveaud et al. 2013; Lee et al. 2020), here we briefly highlight how active shear zones may serve to promote the accumulation and crystallization of economic (i.e., large) pegmatites.

#### Mechanisms of pegmatite melt extraction

The model of anatectic pegmatite formation requires low degrees of partial melting in a fertile protolith to generate melts that are enriched in incompatible elements (Simmons et al. 1995; Shaw et al. 2016; Müller et al. 2017). The threshold for physical interconnectivity of low-volume silicate melts, is often taken to be ~ 7–10 vol%, as determined experimentally (Rosenberg and Handy 2005). However, Etheridge et al. (2021) showed that connectivity may be achieved at lower degrees of melt in actively deforming environments where dehydration melting processes dominate, given the volume increase caused by such reactions may drive sudden brittle failure and allow channelization of melt at proportions as small as 2 vol%. Given reducing the melt volume required for extraction enhances the degree of enrichment and anatectic source is able to achieve, active deformation may play an important role in realizing this model.

Similarly, the model of pegmatite melt extraction from highly fractionated granite systems (either as individual plutons or long-lived mid-crustal magma chambers) is also constrained by melt connectivity, in this case limiting the extent of fractionation that can be invoked prior to system lock-up (Vigneresse and Tikoff 1999; Koopmans et al. 2024). Although localized overpressure of melt can drive the ejection of melt from granitic source regions through dykes (Rubin 1995; Baker 1998), and pegmatitic intrusions formed in this way are frequently seen (e.g., Roda-Robles et al. 2023) these intrusions will be limited in size due to an absence of significant strain anisotropies in the surrounding host rocks (Brisbin 1986). Alternatively, the presence of tectonic stresses within a melt-present environment facilitates efficient extraction of melt (Brown and Solar 1998; Sawyer et al. 2011; Brown 2013), and enable large volumes of highly fractionated melts to migrate out of their granite source region (Černý et al. 2005).

It is thus worth noting that irrespective of the source mechanism invoked, the extraction of large amounts of melt is best facilitated in environments of active deformation. This promotes the formation of fewer, large, intrusions (as observed at Zulu), as opposed to several smaller pegmatitic bodies (e.g., pegmatitic pockets internal to –, or radial pegmatite haloes around the source granite).

#### Pegmatite melt migration

Upon extraction of the final pegmatitic melt from the source region, the style and character of pegmatitic melt migration upwards through the crust before reaching a trap depends on host rock rheology, temperature, and strength anisotropies within the crust (Brisbin 1986). To promote the necessary undercooling to generate characteristic pegmatitic textures (Černý et al. 2005; London 2005; Simmons and Webber 2008; McCaffrey and Jowitt 2023), pegmatitic melts need to be rapidly extracted from their source region and emplaced into cooler host rocks, or chemically quenched during ascent. If melt is present during ductile shearing, then these shear zones can generate regions of increased porosity and dilation, providing a siphon force for melt migration (Sibson et al. 1975; Etheridge et al. 2021), which is an effective way of forcing large volumes of melt to migrate through the crust (Brown 2013).

**Fig. 13:**
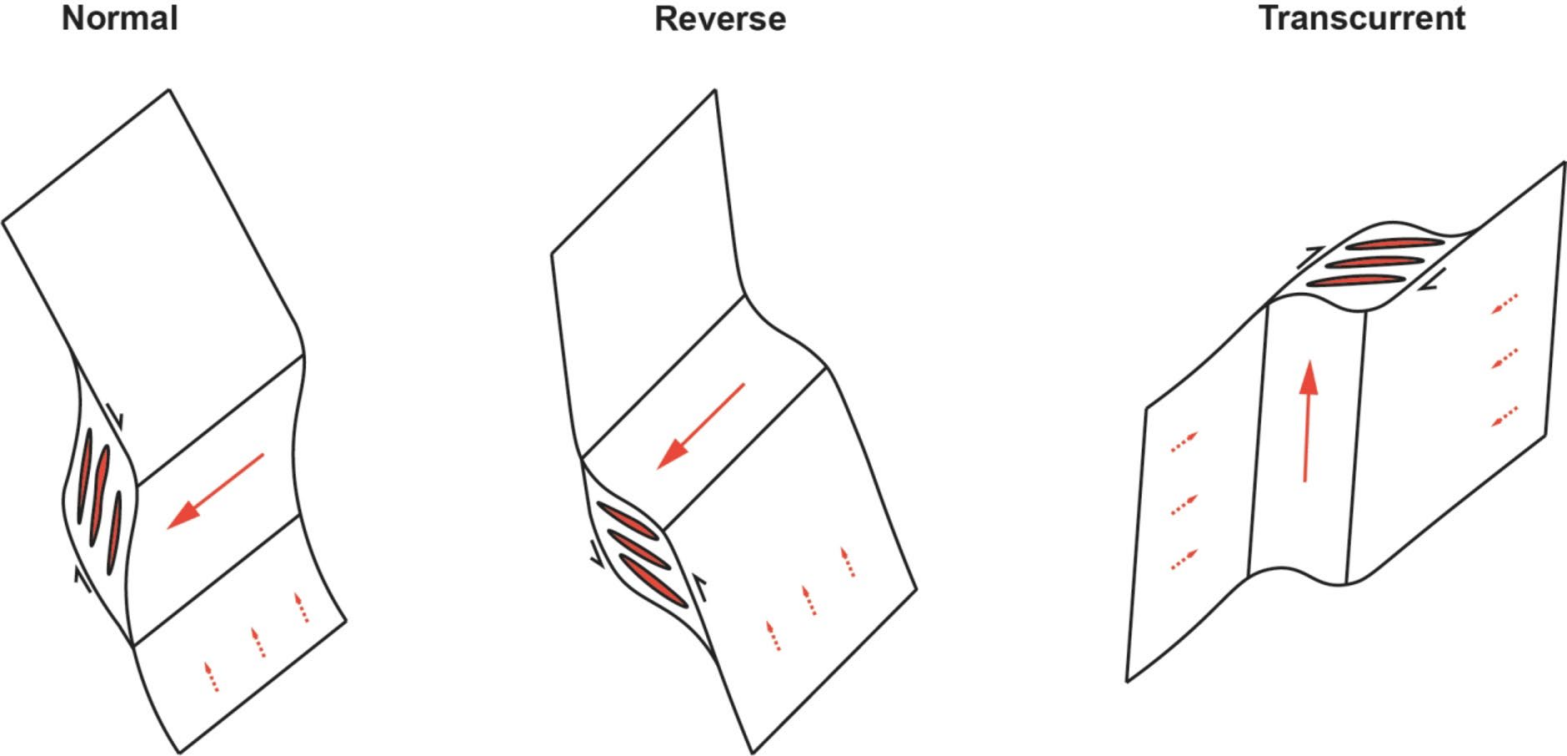
Schematic sketches of melt flow orientations relative to three major structural types (modified from Etheridge et al. 2021). Dashed lines represent preferred direction during percolative flow, and solid lines represent preferentially direction of channelized flow. The geometry of dilational jogs is shown for each fault type. Both normal and reverse faults generate subhorizontal zones of dilation, whereas transcurrent systems generate vertical conduits.

The type of shear-zone system (transcurrent, normal, reverse, or oblique) also has an effect on the vector of melt flow (Fig. 13, Brisbin 1986; Brown 2013; Etheridge et al. 2021). Channelized flow along vertical dilatant conduits preferentially occurs in both transcurrent and reverse shear zones, and are therefore favourable targets for melt migration (Fig. 13, Lindroos et al. 1996; Brown and Solar 1998). Furthermore, transcurrent shear zones (such as the ILSZ) will promote a horizontal percolation of flow during shearing, enabling the concentration of melt from a large melt region towards sites of lower pressure prior to vertical extraction, enabling tapping of a large fertile source region (Fig. 13, Sibson et al. 1975; Brown and Solar 1998; Cavalcante et al. 2016). In this way, transcurrent releasing bends are highly favorable sites of pegmatitic melt migration and subsequent emplacement, given a fertile source is intersected, such as at Zulu or the examples presented above.

Importantly, during the waning stages of shearing, pre-existing anisotropies caused by lithological boundaries, fractures, or tensile cracks, will preferentially allow residual melt migration through the system as stress is relieved. This will result in a change of preferred orientation of late-kinematic pegmatites to oblique to the controlling structure, as is seen with Type 2 pegmatites in the Zulu pegmatite field (Brisbin 1986; Araújo et al. 2001; Demartis et al. 2011; Bhatt et al. 2019). Given sufficiently high melt pressure and vertical flow, late pegmatitic melts may generate large intrusions (e.g., the Tanco deposit) into higher-order late brittle fractures or tension gashes (Brisbin 1986; Duguet et al. 2009; Kremer 2010; Bhatt et al. 2019). In this scenario, pegmatites of economic size may not be orientated parallel to the trend of the major shear zone, as subordinate structures can form oblique to the major shear zone strike (Figs. 10 and 11).

#### Implications for exploration strategies

Irrespective of the source of pegmatites, we suggest that crustal shear zones can play a fundamental role in allowing pegmatitic melt to migrate away from its source region. The localization of melt within shear zones enables the formation of large bodies of evolved melt, in contrast to smaller individual bodies formed around a parental granite in the absence of tectonic stresses (Černý 1991). Thus, when present, shear zones act as a first-order control on the spatial distribution of large pegmatite fields, as is seen at several major deposits such as Greenbushes, Tanco, and Zulu.

We propose that desktop studies should focus on delineating potential structural corridors through which pegmatitic melts may have migrated, with a preference for transcurrent shear zones. The presence of granites with fertile characteristics (reduced K/Rb, elevated concentrations of incompatible elements such as Li, Rb, F, Cs, Selway et al. 2005) along the shear zone may further indicate that the shear zone has intersected a fertile source region, though does not imply that the fertile granites are the source of pegmatites. Such a systematic approach can help delineate the new prospective regions for discovering economic pegmatite bodies.

## Conclusions

The Zulu pegmatite field is an excellent case study with which to highlight the importance of ductile shear-related deformation on both the emplacement mechanisms of pegmatites – where they control the orientation, distribution, and width of individual bodies – alongside controlling the degree of subsolidus recrystallization a pegmatite experiences during deformation. At Zulu, major competency contrasts between lithologies localized high-strain domains during sinistral strike-slip shearing. Pegmatites were emplaced into subparallel dilational jogs syn-kinematic to shearing and subsequently extensively recrystallized during continued deformation, driving the (re)precipitation of fine-grained spodumene and muscovite. By contrast, late-kinematic pegmatites at Zulu are emplaced along late-kinematic subordinate fracture sets and tension gashes oblique to the principal shearing direction and preserve their magmatic mineralogy owing to limited subsolidus deformation.

Shear zones are known to be regions that allow efficient migration of silicate melt through the continental crust. In the presence of a fertile source, specifically transcurrent shear zones enable efficient migration of significant pegmatitic melt from a hot source region to a shallower level, cooler host rock, driving undercooling and enabling the rapid crystallization textures distinctive of pegmatites. Future greenfields exploration should therefore focus on constraining transcurrent shear zones where significant strength anisotropies are present, with indicators of fertility at the melt source (e.g., enriched granites along the shear zone). Constraining the structural history and relative timing of pegmatite emplacement through detailed fieldwork and petrographic analyses can subsequently shed light on the distribution and orientation of individual pegmatite bodies within a pegmatite field. Importantly, a focus on constraining the source (granite vs. anatectic) of a pegmatite district may therefore be of lesser importance, as regional structures have a more important control on the distribution of individual bodies within a district.
